# Comparative transcriptome profiling of a resistant vs susceptible bread wheat (*Triticum aestivum* L.) cultivar in response to water deficit and cold stress

**DOI:** 10.7717/peerj.11428

**Published:** 2021-05-12

**Authors:** Dmitrii K. Konstantinov, Ulyana S. Zubairova, Anton A. Ermakov, Alexey V. Doroshkov

**Affiliations:** 1 The Federal Research Center Institute of Cytology and Genetics, Siberian Branch of the Russian Academy of Sciences, Novosibirsk, Russian Federation; 2 Novosibirsk State University, Novosibirsk, Russian Federation

**Keywords:** Bread wheat (*Triticum aestivum* L.), Saratovskaya 29, Yanetzkis Probat, Cold stress, Water deficiency stress, Massive analysis of cDNA ends (MACE), Transcriptome profiling, Bioinformatics, Systems biology

## Abstract

Bread wheat (*Triticum aestivum* L.) is one of the most important agricultural plants wearing abiotic stresses, such as water deficit and cold, that cause its productivity reduction. Since resistance to abiotic factors is a multigenic trait, therefore modern genome-wide approaches can help to involve various genetic material in breeding. One technique is full transcriptome analysis that reveals groups of stress response genes serving marker-assisted selection markers. Comparing transcriptome profiles of the same genetic material under several stresses is essential and makes the whole picture. Here, we addressed this by studying the transcriptomic response to water deficit and cold stress for two evolutionarily distant bread wheat varieties: stress-resistant cv. Saratovskaya 29 (S29) and stress-sensitive cv. Yanetzkis Probat (YP). For the first time, transcriptomes for these cultivars grown under abiotic stress conditions were obtained using Illumina based MACE technology. We identified groups of genes involved in response to cold and water deficiency stresses, including responses to each stress factor and both factors simultaneously that may be candidates for resistance genes. We discovered a core group of genes that have a similar pattern of stress-induced expression changes. The particular expression pattern was revealed not only for the studied varieties but also for the published transcriptomic data on cv. Jing 411 and cv. Fielder. Comparative transcriptome profiling of cv. S29 and cv. YP in response to water deficit and cold stress confirmed the hypothesis that stress-induced expression change is unequal within a homeologous gene group. As a rule, at least one changed significantly while the others had a relatively lower expression. Also, we found several SNPs distributed throughout the genomes of cv. S29 and cv. YP and distinguished the studied varieties from each other and the reference cv. Chinese Spring. Our results provide new data for genomics-assisted breeding of stress-tolerant wheat cultivars.

## Introduction

Wheat is one of the most important crops in the world (Gill et al., 2004), which provides between 13% and 57% of calories consumed by people, depending on the country (Portmann, Siebert & Döll, 2010; Farook et al., 2018). However, several abiotic factors, such as fluctuations in temperature, drought, and the content of minerals in the soil, affect its productivity (Chaves, Maroco & Pereira, 2003; Chaves, Flexas & Pinheiro, 2009; Ashraf & Harris, 2013). It was showed that heat and drought occur to a loss of up to 40% of the crop (Zampieri et al., 2017); exposure to cold leads to a decrease in the mass of 1,000 grains and the number of grains per plant (Subedi et al., 2000; Ji et al., 2017). Moreover, a prolonged stressful effect leads to significant crop losses (Ji et al., 2017). Thus, breeding stress-resistant wheat varieties leads to more stable crops. Identification of candidate genes and their validation is an essential step for selection aimed at obtaining the required agricultural traits (Sehgal, Baliyan & Kaur, 2019).

It is well known that drought tolerance is a polygenic and sophisticated feature that includes maintaining the stable functioning of the photosynthetic apparatus, as well as ionic and water homeostasis in tissues, nutrient metabolism, and regulation of plant growth. Currently, for bread wheat, a number of quantitative traits loci (QTL) markers were identified under conditions of water deficiency (Mwadzingeni et al., 2016; Muqaddasi et al., 2019); these markers are also responsible for the content of chlorophyll (Czyczyło-Mysza et al., 2011; Ilyas et al., 2014; Tahmasebi et al., 2016), water use efficiency (Parent et al., 2015), plant height, number and weight of grains for various drought stress variants (Tarawneh et al., 2019; Liu et al., 2019). However, the QTL approach often highlights large chromosome regions (more than 10 cM) containing over 100 genes (Pandit et al., 2010; Iquebal et al., 2019). Also, the introgression of large chromosome fragment not always leads to the expected phenotype (Hospital, 2005). Therefore, it is essential to use specific genes that provide the QTL effect instead of QTL itself (Ron & Weller, 2007).

Gene expression profiling allows identifying candidate genes for targeted breeding. Full-genome measurements of gene expression under drought conditions were carried out by various researchers using microarrays (Qin et al., 2008; Aprile et al., 2009; Li et al., 2012) and RNA-seq (Lv et al., 2018; Iquebal et al., 2019). It was shown that in drought conditions, wheat activates the metabolism of sucrose and starch (Poersch-Bortolon et al., 2016). Further studies revealed that drought response varies between different genetic lines (Hu et al., 2018). In wheat, drought results in an increase in the expression of genes associated with the pathways of biosynthesis abscisic acid (ABA) and jasmonic acid, as well as the transmission of these hormonal signals (Wang et al., 2020). Also, lipid metabolism (‘lipid and fatty acid biosynthesis,’ and ‘fatty acid metabolic process’) was shown to play an essential role in the response of wheat to drought after heavy metal (molybdenum) exposure (Wu et al., 2020). Besides that, exposure to exogenous spermidine increases the activity of superoxide dismutase (SOD), peroxidase (POD), and catalase (CAT) in drought conditions in spike grains (Li et al., 2020). Genes associated with the antioxidant system and osmotic stress response had a higher expression level for the drought-tolerant cv. JM-262 than for the drought-sensitive cv. LM-2 (Hu et al., 2018). It was shown that differentially expressed genes (DEGs) are associated with flowering, the functioning of stomata, and the photosynthetic apparatus (Ma et al., 2017). Investigating drought stress under field agronomic conditions on cv. Chinese Spring showed the enrichment of stress-related genes at the distal ends of chromosomes compared to the proximal regions in the wheat genome (Gálvez et al., 2019). Another group studied changes in gene expression in response to low-temperature conditions (Zhao et al., 2019). They showed an increased expression of genes associated with dehydrin and proteins of late embryogenesis, as well as genes encoding transcription factors, such as genes with leucine repeats and EF-hand proteins, which regulate the efficiency of transcription of downstream protective genes. It was revealed that cold stress on durum wheat causes a decrease in the expression of Glutaredoxin genes, proteins associated with the calcium signal, and an increase in the expression of hormonal signal transduction genes (Díaz et al., 2019). However, in these works, the data were not compared with QTL. It should be noted that wheat varieties from different regions differ in responses to stress. Furthermore, for polyploid plants, homeoallelic genes might differ in contribution to the abiotic stress response. For example, for allopolyploid cotton (*Gossypium hirsutum* L.), one copy of alcohol dehydrogenase A (AdhA) changes its expression under waterlogging, and the other changes its expression when exposed to cold (Liu & Adams, 2007). Bread wheat is an allohexaploid containing three subgenomes (Brenchley et al., 2012). Its homeoallelic genes are shown to have different expression patterns in normal conditions (Bottley & Koebner, 2008; Leach et al., 2014) and under salt stress (Zhang et al., 2016).

Low-temperature tolerance may differ between spring and winter wheat depending on the allelic state, the vernalization loci (Vrn-1) on the group 5 chromosomes. For cold-hardy winter wheat (Norstar × Winter Manitou), additional loci (QTL) were found: a strong one on chromosome 5A, a weak one on chromosome 1D, and numerous loci with more low probability values (Båga et al., 2007). The 5A QTL located 46 cM proximal to the vrn-A1 locus explained 40% of the cold tolerance variance. The authors indicate two C-repeat Binding Factor (CBF) genes as major candidates. There is reason to believe that the observed varying degrees of tolerance to low temperatures are due to many genes (Gorji, Hajianfar & Rostamforody, 2014). Thus, the most large-scale studies using GWAS also confirm the major locus on chromosome 5A, but they reveal 76 significant SNPs associated with cold resistance (Zhao et al., 2020). These significant SNPs were scattered over 18 chromosomes, resulting in 361 candidate genes. Primary enrichment is associated with various events of regulation and amino acid metabolism.

Resistant and susceptible bread wheat varieties differ in gene expression patterns in response to stress (Hu et al., 2018). In this regard, the most useful and vital objects for a genome-wide analysis are the most evolutionary distant varieties. We performed gene expression profiling in leaves for two varieties of bread wheat from European and Russian breeding in this work. Cv. Saratovskaya 29 (S29) from the Saratov breeding station is a typical long-stemmed, drought-tolerant variety obtained for the northern and temperate latitude zones (Kumakov, Evdokimova & Buyanova, 2001; Mollasadeghi et al., 2011; Mollasadeghi, 2012). This variety is widely used in agronomy and is characterized by high-quality flour (Laikova et al., 2004). Cv. Yanetzkis Probat (YP) from East Germany is significantly more sensitive to drought than cv. S29 resulting in lower yields under stress conditions (Osipova et al., 2013). In this work, for cv. S29 and cv. YP, we conducted the first targeted sequencing of protein-coding transcripts in leaves from plants grown under a set of abiotic stress conditions (water deficiency (WD) and exposure to cold for six and 24 h, and also under favorable conditions). For both varieties, we found out groups of genes involved in the transcriptional response to cold and WD stress and identified a core group of coordinately functioning genes with a uniform pattern of stress-induced changes in expression.

These genes revealed the same behavior for some other wheat varieties (cv. Jing 411 and cv. Fielder) involved in the additional meta-analysis. For cv. S29 and cv. YP, we confirmed the hypothesis suggested in (Gálvez et al., 2019) about homoeologous genes’ subfunctionalization under stress conditions. Our data also confirmed a relatively lower variation in expression of genes related to the same homoeologous group if at least one changed significantly in response to stress. The data also revealed several SNPs distributed throughout the genomes and distinguished the studied varieties from each other and the reference cv. Chinese Spring (CS). These findings will allow us to develop new markers and create mapping populations based on the studied varieties. It is incredibly valuable for studying the collection of lines with the substitution of individual chromosomes of the recipient cv. S29 with the donor cv. YP’s homologous chromosomes (Trufanov et al., 2007; Pestsova et al., 2000) showing high polymorphism in the traits associated with the stress response. Therefore, this work is an essential step for expanding the spectrum of candidate genes and involving new material for genetic research and genomic selection.

## Materials and Methods

### Plant material, stress treatments, and sampling

The hexaploid *Triticum aestivum* L. cultivars Saratovskaya 29 (S29, a resistant cultivar) and Yanetzkis Probat (YP, a susceptible cultivar) (Doroshkov, Pshenichnikova & Afonnikov, 2011; Doroshkov et al., 2016) were subjected to transcriptome analyses upon the standard, water deficiency (WD) and low-temperature conditions. Germinated seeds for both cultivars were planted in plastic containers with drainage (PET, size 56 × 39 × 28 cm) filled with expanded clay balls. In each container, six plants of every variety were grown up to the four-leaf stage. Plants were grown in a greenhouse (Laboratory of Artificial Plant Growth at the Institute of Cytology and Genetics SB RAS, Russia) under an 18/6 h photoperiod (artificial supplementary lighting by Sylvania SHP-TS 600 w, illumination of plants was carried out as 15 − 20 × 10^3^4 lx); the temperature condition was maintained at 16–18 °C at night and 20–23 °C during the day. Watering with Knop solution (Hershey, 1994) was carried out daily until complete saturation of the substrate. Knop solution with additional microelements was used for watering once a week.

Cold stress was simulated in a climatic chamber at +4 °C and the same day/night lighting and watering conditions as in a greenhouse during 6 h (short-term stress) and 24 h (long-term stress).

We simulated WD stress by stopping the watering of the substrate for two weeks. Preliminarily we measured the moisture content of the expanded clay in a dried and fully saturated by Knop solution state with a substrate moisture meter (model MG-44, Vesoizmeritel company, Arzamas, Russia). A calibration curve reflecting the relative degree of the substrate saturation with water was constructed (values of clay humidity are given in File S1). As a 100% humidity level, we considered the moisture retained by expanded clay after prolonged steeping in water; as a 0% humidity level, we considered the moisture of expanded clay dried in the open air for 2 weeks and then in an oven at 200 °C for 2 h. It should be noted that plant roots cannot take away all water retained in expanded clay, and at least a 50% humidity level corresponds to WD stress conditions, which was achieved within 2 weeks after cessation of substrate wetting.

**Figure 1:**
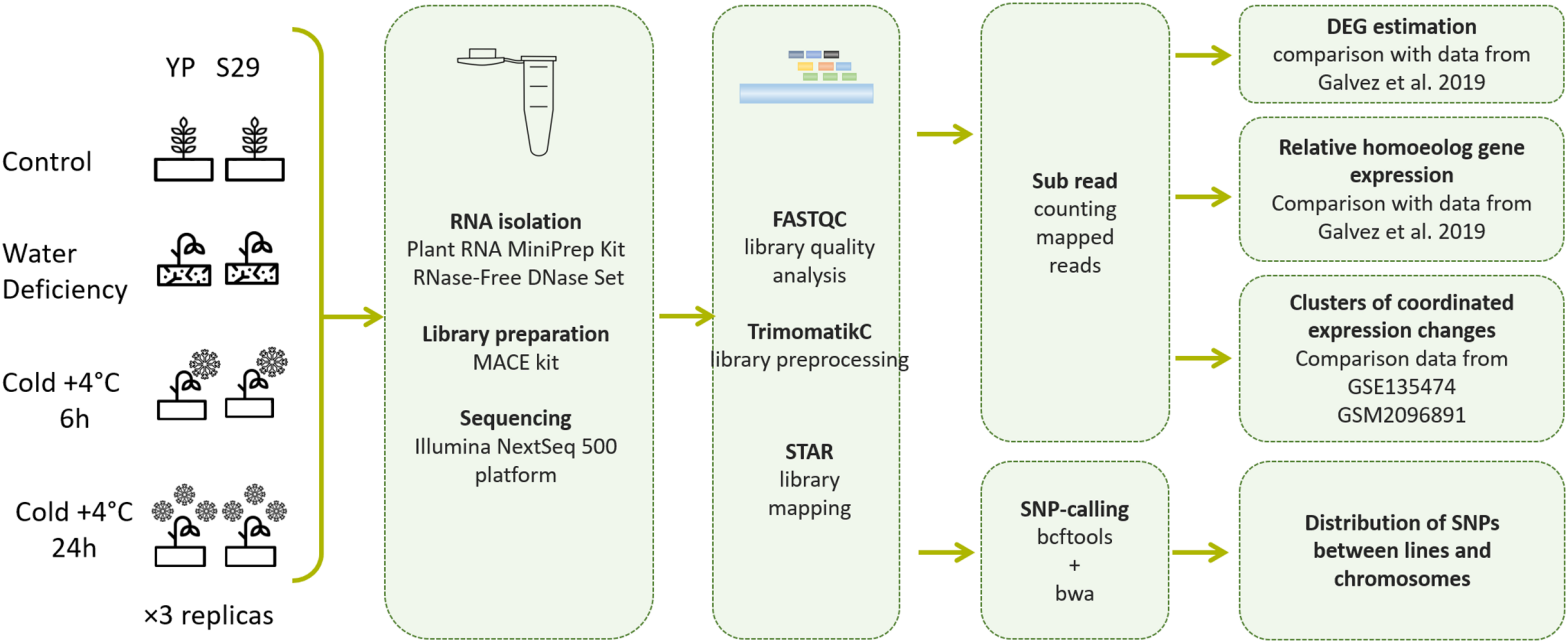
Pipeline of experimental methods and data processing methods used in this work.

All experiments (short-term 6 h cold stress, long-term 24 h cold stress, and WD stress) were performed at the same time, and seedlings grown in normal conditions (22 °C and well-watered) were taken as control (Fig. 1). We used samples obtained by mixing the middle parts of the third leaves from three plants of the same variety grown in one container. The total weight of each sample was 100–200 mg. Three samples were collected for each experimental group and immediately frozen in liquid nitrogen and then stored at −70 °C until RNA isolation.

### RNA isolation, library preparation, and transcriptome sequencing

The total RNA from samples was extracted using Plant RNA MiniPrep Kit (Zymo Research, Irvine, CA, USA) with additional treatment with RNase-Free DNase Set (QIAGEN, Hilden, Germany) according to the manufacturer’s instructions and protocols. Evaluation of the quantity and purity of RNA was performed on a Nanodrop 2000 spectrophotometer (Thermo Scientific, Wilmington, DE, USA); integrity was assessed using LabChip GX Touch™ system (PerkinElmer, Waltham, MA, USA).

MACE libraries were constructed using the MACE kit (Zawada et al., 2014) according to the manual provided with the kit. Sequencing was performed on Illumina NextSeq 500 platform. PCR duplicates were removed automatically.

All raw data are available in NCBI SRA (PRJNA630059).

### Bioinformatic analysis

To analyze the libraries’ quality, we used the FastQC program. Trimming was performed using trimmomatic (Bolger, Lohse & Usadel, 2014) with the following parameters:

CROP:63 HEADCROP:13 SLIDINGWINDOW:4:18 MINLEN:42

Reads containing poly-N sequences and low-quality reads were removed from the raw data to obtain clean reads. Libraries were cleaned using the bbduk from the bbmap software package in order to remove readings containing a high proportion of adenines. Q20 and Q30 values and sequence duplication levels were determined following conventional methods. The further analysis used clean reads with high quality. Wheat reference genome and genome markup file (gff-file) from URGI database (Alaux et al., 2018) was used. Reads were mapped to the reference genome using STAR (Dobin et al., 2013) with the following parameters:

STAR --runThreadN 24 --genomeDir db_wheat --readFilesIn $file --outFileNamePrefix /star_mup/$file/ --outReadsUnmapped Fastx

The featureCounts program from the subread-1.6.3-source package was used to count the reads mapped to each gene (Liao, Smyth & Shi, 2013). The FPKM metric was used to evaluate gene expression levels; however, due to the MACE technique’s peculiarities, normalization of the gene length was not performed. The following algorithm was used to identify differentially expressed genes (DEGs). At the first stage, genes with an expression level of less than 0.5 in each experimental point were filtered (the threshold was taken from (Gálvez et al., 2019)). At the second stage, we selected genes with more than doubled expression between control and stressful conditions. At the third stage, a t-test was performed, taking into account the correction for multiple Benjamini–Yekutieli comparisons (FDR = 0.05). Thus, we generated a set of DEGs changing expression for at least one variety and at least in one experimental point. Searching of genes with a correlated expression pattern and visualization of gene expression was performed using the matplotlib library in Python 3.6.

For the set of DEGs, differences in the expression levels in stress relative to the control conditions were calculated for each treatment and each variety. Each of the obtained values was assigned to one of six classes: (i) decreased more than two times, (ii) decreased less than two times, (iii) almost unchanged, (iv) increased less than two times, (v) increased more than two times, (vi) increased more than five times. In total, there are 46,656 variants of combining such classes for a tuple (YP WD, YP cold 6 h, YP cold 24 h, S29 WD, S29 cold 6 h, S29 cold 24 h), but only a small part of them turned out to be populated with DEGs. We selected the 20 most populated classes, including DEGs that consistently change expression in both varieties in response to WD and cold stress during 6 h and 24 h. For these classes, we compared the change of gene expression for cv. S29 and cv. YP and revealed 43 genes that change expression in response to stresses in the same way. For these genes, we examined changes in expression in datasets for cv. Jing 411 in cold acclimation (according to GSE135474) and cv. Fielder in drought conditions (according to GSM2096891). The correlation of gene expression for replicas visualized via heatmap, and additional principal component analysis (PCA) were evaluated using the matplotlib and sklearn libraries in Python 3.6. The intersection of gene lists and Venn diagrams’ visualization were performed using the Venny 2.1 online tool.

The description of gene functions was determined using the URGI database, access date 2020.01.15 (Alaux et al., 2018). To search for the orthologous genes for *Arabidopsis thaliana* L., BLASTp (e-value < 0.0001) was performed. The description of *A.thaliana* genes was taken from the TAIR database (access date 2020.01.15).

Identification of domains in proteins was carried out using the hmmsearch program of the package HMMER v.3 and the Hidden Markov Models (HMM) taken from the Pfam database using the threshold e-value = 1e−7.

Additionally, for functional annotation, we used the following tools g:Profiler (Reimand et al., 2007), AgriGo (Tian et al., 2017), to evaluate the associative map of GO terms, we used AmiGO 2 (Carbon et al., 2009).

To identify homeoallelic gene sets, we used BLASTp (e-value < 0.0001, identity > 0.6, target = 3) for the bread wheat proteome. We selected genes with target labels located on three subgenomes. The homeoallelic gene sets were divided into seven classes according to the following algorithm described by Gálvez et al. (2019). (i) “A- B- or D-dominant” classes were selected if the ratio of corresponding copy expression was greater than 66%, then (ii) “A- B- or D-suppressor” classes were selected if the ratio of corresponding copy expression was less than 16%, and (iii) the others were grouped into the “balanced” class.

In order to identify SNPs, the results of reads mapping obtained by STAR were converted to a VCF format using bcftools v1.5. A version of the genome from URGI database (Alaux et al., 2018) was considered the reference genome (see details above). SNPs with an indicator QUAL > 30 were considered as reliable. For functional annotation of SNPs, the cf-file was transferred to SnpEff v4.3 (Cingolani et al., 2012). To select SNPs that are homozygous for the alternative allele in the three wheat genomes, we used Python 3.6 scripts.

## Results

### Stress-induced full transcriptome data for bread wheat cultivars Saratovskaya 29 and Yanetzkis Probat

Full transcriptome data for bread wheat (*Triticum aestivum* L.) cv. Saratovskaya 29 (S29) and cv. Yanetzkis Probat (YP) grown under short cold (6 h), long cold (24 h), and water deficiency (WD) conditions were obtained using Illumina based MACE technology. After removing barcodes and reads with low quality of reading and with poly-A enrichment, we revealed the following data characteristics: (i) the quality of reading nucleotides was more than 25 on average; (ii) the reading length was 62–69 pairs of nucleotides; (iii) The library accounted for approximately 7.2 million reads (from 4.8 to 12.4 million); (iv) the nucleotide content of GC pairs is 46%; (v) adapter sequences were not detected. It should be noted that the amount of adenine increases slightly from 5′ to 3′ ends at the reads. MACE’s methodological specificity might cause this effect because, for some transcripts, the reading of the poly-A tail occurred instead of the protein-coding sequence. On average, 49.6% of readings were mapped uniquely to the genome, and about 15% of readings had from 2 to 4 mapping locations, which probably belong to homeoallelic genes on different chromosomes. For all samples, the summary of mapping on the wheat genome is given in a File S2.

**Figure 2:**
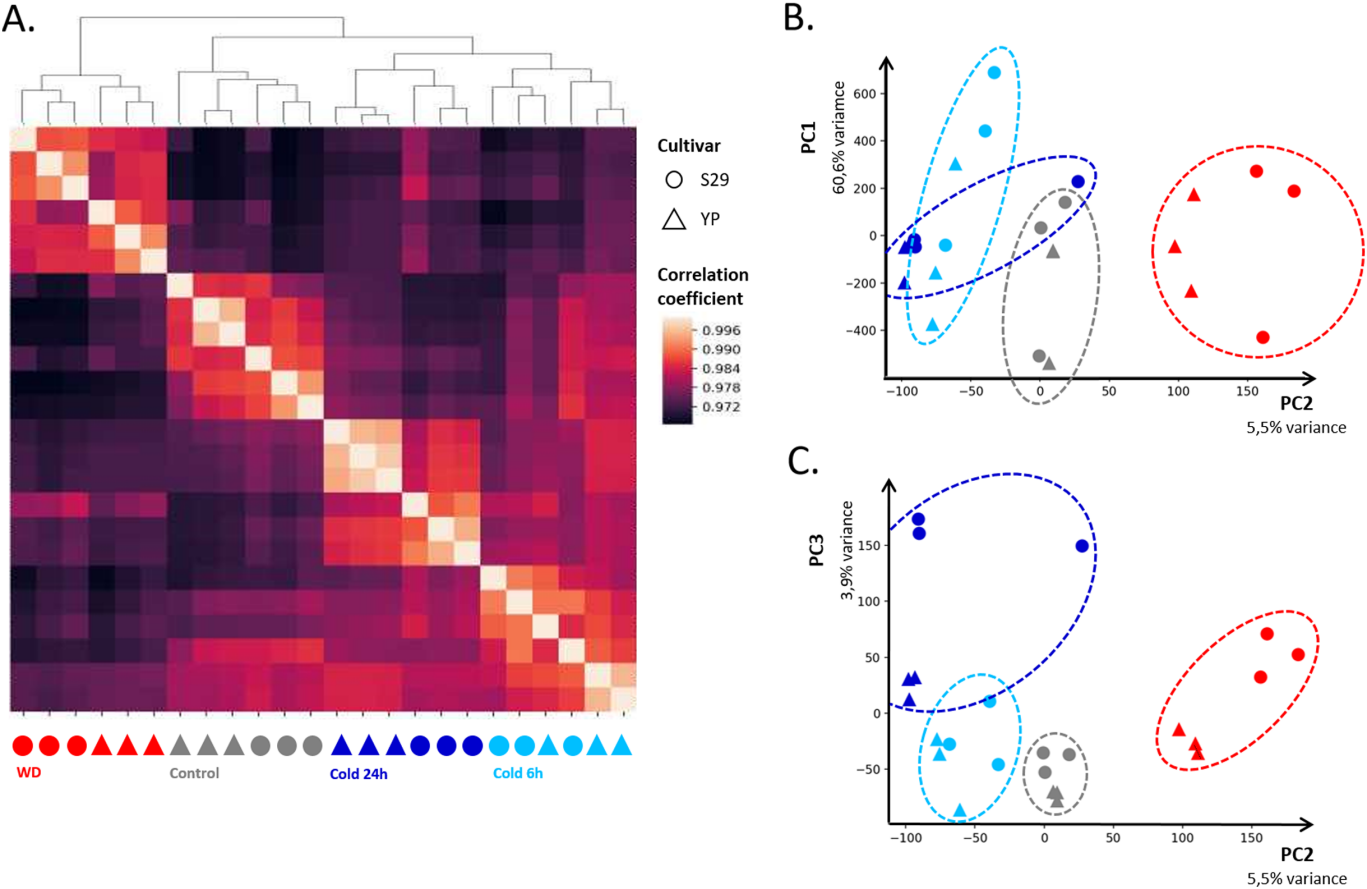
Clustering of sequenced libraries shows similar homogeneous replicas on dendrogram and heatmap (A), as well as on scatter plots for the first and second (B) and second and third (C) principal components. PC1, PC2, and PC3 respectively accounted for 60.6%, 5.5%, and 3.9% of the observed variation.

In order to assess the consistency between biological replicates, Pearson correlation coefficients were calculated for each group. The correlation coefficients between three biological replicates for each of the varieties and each of the growing conditions lied in the range from 0.96 to 0.99. The heat map of correlations between libraries and their clustering (Fig. 2A) shows high convergence of replicas and speaks in favor that this dataset is suitable for further analysis. The heat map (Fig. 2A) indicates that the “Control”, “WD” and “Cold 24 h” clusters have a high correlation within and relatively low correlation with other clusters. The “Cold 6 h” cluster is slightly more correlated with “Cold 24 h” and “Control,” which is consistent with the fact that part of the cold response genes changes its expression only after prolonged exposure to cold. For biological replicates, PCA showed that the first four components describe 60.6, 5.5, 3.9, and 2.7% variance, respectively (Figs. 2B and 2C). It should be noted that, for the first component, the points corresponding to control replicas split up the points corresponding to WD and cold ones, indicating a specific change in gene expression in response to those stresses.

### Differentially expressed genes in response to cold and WD

**Figure 3:**
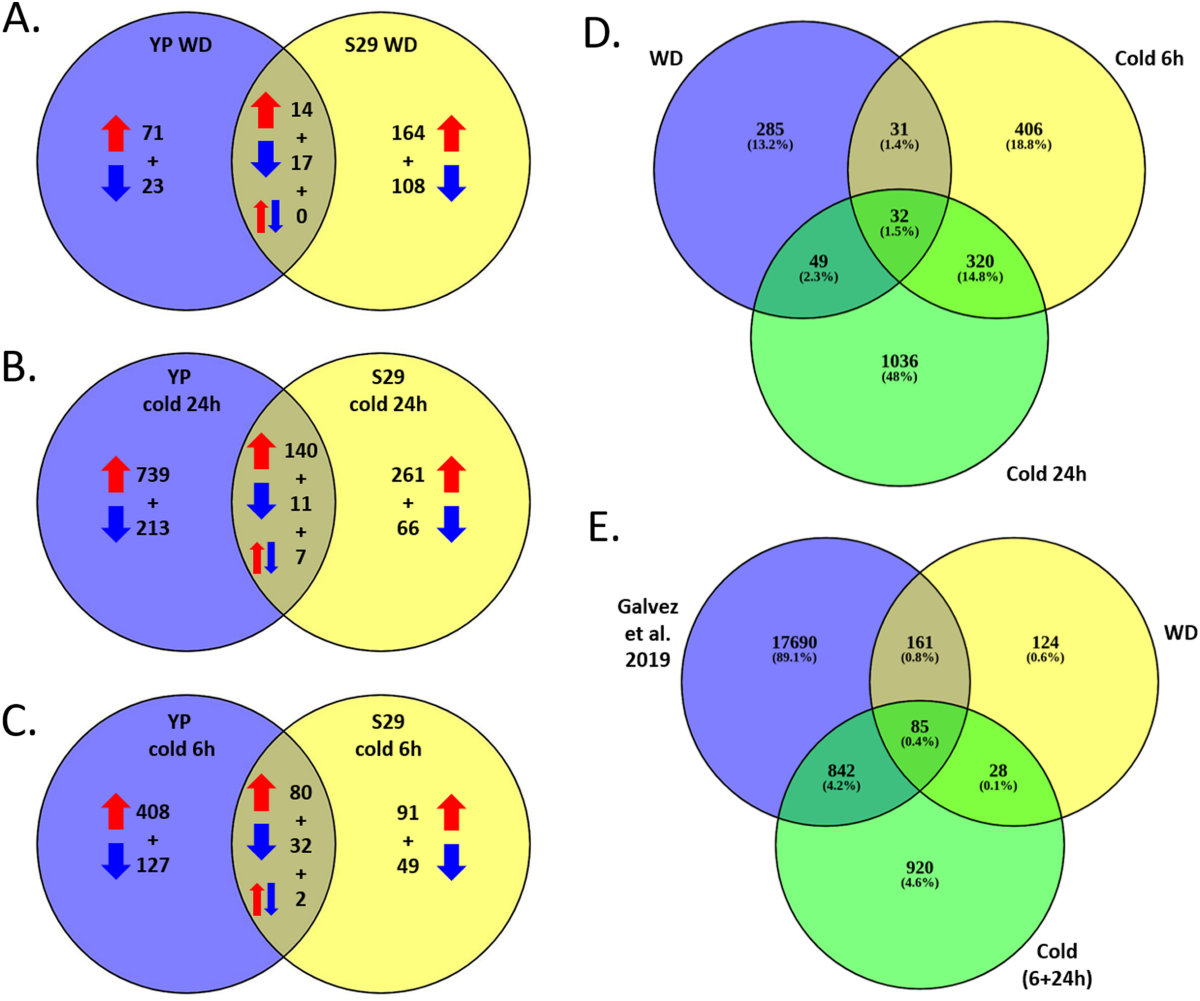
(A–C) Venn diagrams for cv. S29 and cv. YP lists of DEGs corresponding WD (A), long cold (B), and short cold (C) conditions. Red arrows are for up-regulation, blue arrows are for down-regulation. (D) Venn diagrams for WD, short cold stress 6 h, long cold stress 24 h lists of DEGs. (E) Venn diagrams for WD and cold lists of DEGs and a lists of DEGs obtained in (Gálvez et al., 2019).

As a result of full transcriptome data analysis, we identified 2,159 differentially expressed genes (DEGs) that alter expression under stressful conditions as compared to the control (FC > 2). Expression levels within each variety were processed separately, adjusted for multiple comparisons. A list of DEGs and levels of significant changes in expression are given in Files S3 and S4. Figures 3A–3C shows Venn diagrams for lists of DEGs that change expression for two studied varieties under cold and WD stressful effects. It should be noted that the lists of cold and WD response DEGs differ significantly between the cold-tolerant cv. S29 and the cold-sensitive cv. YP. Among 2,159 DEGs for cv. S29, 929 genes undergo a stress-induced expression change, 1,577 genes do for cv. YP, and 347 (16.1%) genes do for both varieties. For the WD-response, 397 genes significantly change expression; for cv. YP, there are 125 genes, for cv. S29, there are 303 genes, while only 31 genes (approximately 7.8%) change expression for both varieties (Fig. 3A). For the 24 h cold-response, 1,437 genes significantly change expression, for cv. YP, there are 1,110 genes; for cv. S29 there are 485 genes, while only 158 genes (approximately 11%) change expression for both varieties (Fig. 3B). For the 6 h cold-response, 789 genes significantly change expression, for cv. YP, there are 649 genes, for cv. S29, there are 254 genes, while only 114 genes (approximately 14.5%) change expression for both varieties (Fig. 3C). In the experiment carried out by Gálvez et al. (2019), 18,778 genes showed differential expression in a drought stress experiment for wheat. For our data, 927 cold and 246 WD-induced DEGs are the same as in the indicated experiment (Figs. 3D, 3E).

Simultaneously under **cold and WD conditions** both studied varieties showed **an increased gene expression** for cytochrome P450 (TraesCS3A01G228200, TraesCS3A01G342900, TraesCS1A01G334700, TraesCS1A01G366500) and glucuronyl transferase (UDPGT) (TraesCS5D01G473500, TraesCS5A01G461100, TraesCS7D01G190600, TraesCS3D01G279700, TraesCS6D01G162700, TraesCS7B01G094400); and **a decreased gene expression** for lutaredoxin (TraesCS5B01G048900, TraesCS5B01G049200, TraesCS5A01G045000).

Changes in the expression of protein kinase genes (tyrosine subclass Pkinase Tyr, Pkinase) and Chloroa b-bind were **oppositely directed** for different paralogs. **Under WD conditions**, **cv. YP** was characterized by a decrease in the activity of transmembrane transporters of amino acids, in particular tryptophan and tyrosine permeases, while **cv. S29** was characterized by **an increase** in the activity of proteins of AAA 16 family (ATPases associated proteins TraesCS7D01G023600, TraesCS5B01G394100, TraesCS2D01G250300, TraesCS2B01G268600, TraesCS6B01G235100), F-box (signal and regulatory proteins TraesCS3A01G390000, TraesCS2B01G514900, TraesCS3A01G253800, TraesCS3B01G285700, TraesCS7B01G127300, TraesCS7A01G110500), ABC transporters (ABC tran TraesCS7D01G023600, TraesCS6B01G235100, TraesCS2B01G268600, TraesCS2D01G250300), Alpha/beta hydrolase superfamily (Abhydrolase 3-TraesCS2A01G143500, TraesCS2A01G143300, TraesCS2B01G168300, TraesCS3D01G097100); and **a decrease** in the expression of ZIP transporters (Zinc transporter TraesCS2B01G533800, TraesCS2D01G146800, TraesCS2A01G505500, TraesCS2A01G143400, TraesCS1A01G297400, TraesCS1D01G294000, TraesCS7D01G412900, TraesCS7D01G413400), Ca^2+^-dependent proteins (EF-hand, SPARC Ca^2+^ bdg TraesCS5B01G059900, TraesCS1D01G239000, TraesCS3B01G536200, TraesCS3D01G483200, TraesCS7B01G350200, TraesCS7D01G439800), and leucine-rich repeat.

**Both varieties** demonstrate a decrease in the expression of genes related to the photosynthetic apparatus (RuBisCO small TraesCS2A01G067100, TraesCS2B01G079200, TraesCS2A01G066700).

**Under cold conditions (6 and 24 h)**, both varieties showed **increased gene expression** for proteolipid genes of the cell wall (Pmp3 TraesCS5A01G360000, TraesCS5B01G362500, TraesCS4B01G197300, TraesCS4D01G197700), Ca^2+^-regulated kinases (EF-hand TraesCS4B01G182900, TraesCS6D01G149300, TraesCS6A01G162900); and a **decreased gene expression** for genes associated with chloroplast and thylakoid membranes.

**Under conditions of short cold (6 h)**, **both varieties** demonstrated increased expression of genes related to kinases and B-box zinc finger (zf-B box-TraesCS7A01G490800, TraesCS7D01G477100, TraesCS6A01G153700, TraesCS6D01G143100); **for cv. YP**, genes associated with heavy metals (HMA), glutathione S-transferases (TraesCS1A01G078800, TraesCS7A01G362900, TraesCS1D01G081200) and different subclasses of EF-hand (Ca^2+^-dependent) increased activity.

Sometimes, differences in stress tolerance between varieties are associated with unequal and even opposite gene expression changes that respond to stress. There were no DEGs with these properties under WD-conditions for cv. S29 and cv. YP. However, the transcriptomic data analysis revealed eight genes that decreased expression for cv. S29 and increased expression for cv. YP in response to 6 h and 24 h cold exposure (Table 1). For long-term cold stress (24 h), seven genes were identified, including three genes coding transcription factors (two WRKY genes among them), calmodulin-like protein, a transmembrane protein, and the late embryogenesis abundant gene, and a gene with unknown function. For short cold stress (6 h), we identified the proline-rich nuclear receptor coactivator gene. For cold conditions, we revealed no genes that increased expression for cv. S29 and decreased expression for cv. YP.

**Table 1:** List of DEGs significant decreasing expression for cv. S29 and increasing expression for cv. YP in response to cold stress (E-value < 0.05 after Benjamini–Yekutieli correction).

Cold expose	*T. aestivum* gene	*A. thaliana* ortholog	Description according to TAIR database
24 h	TraesCS2A01G583500	AT2G35460	Late embryogenesis abundant (LEA) hydroxyproline-rich glycoprotein family
	TraesCS4D01G063900	AT3G57450	hypothetical protein
	TraesCS5A01G185700	AT2G46400	Encodes a WRKY transcription factor that contributes to the feedforward inhibition of osmotic/salt stress-dependent LR inhibition via regulation of ABA signaling and auxin homeostasis.
	TraesCS6D01G136200	AT1G80840	Pathogen-induced transcription factor. Binds W-box sequences in vitro. Forms protein complexes with itself and with WRKY40 and WRKY60. Coexpression with WRKY18 or WRKY60 made plants more susceptible to both *P. syringae* and *B. cinerea*. WRKY18, WRKY40, and WRKY60 have partially redundant roles in response to the hemibiotrophic bacterial pathogen *Pseudomonas syringae* and the necrotrophic fungal pathogen *Botrytis cinerea*, with WRKY18 playing a more important role than the other two. The mRNA is cell-to-cell mobile.
	TraesCS5B01G059900	AT3G22930	Encodes a calmodulin-like protein.
	TraesCS5D01G235600	AT2G31945	transmembrane protein
	TraesCS2A01G476000	–	–
6 h	TraesCS5D01G270400	AT3G21570	proline-rich nuclear receptor coactivator

**Figure 4:**
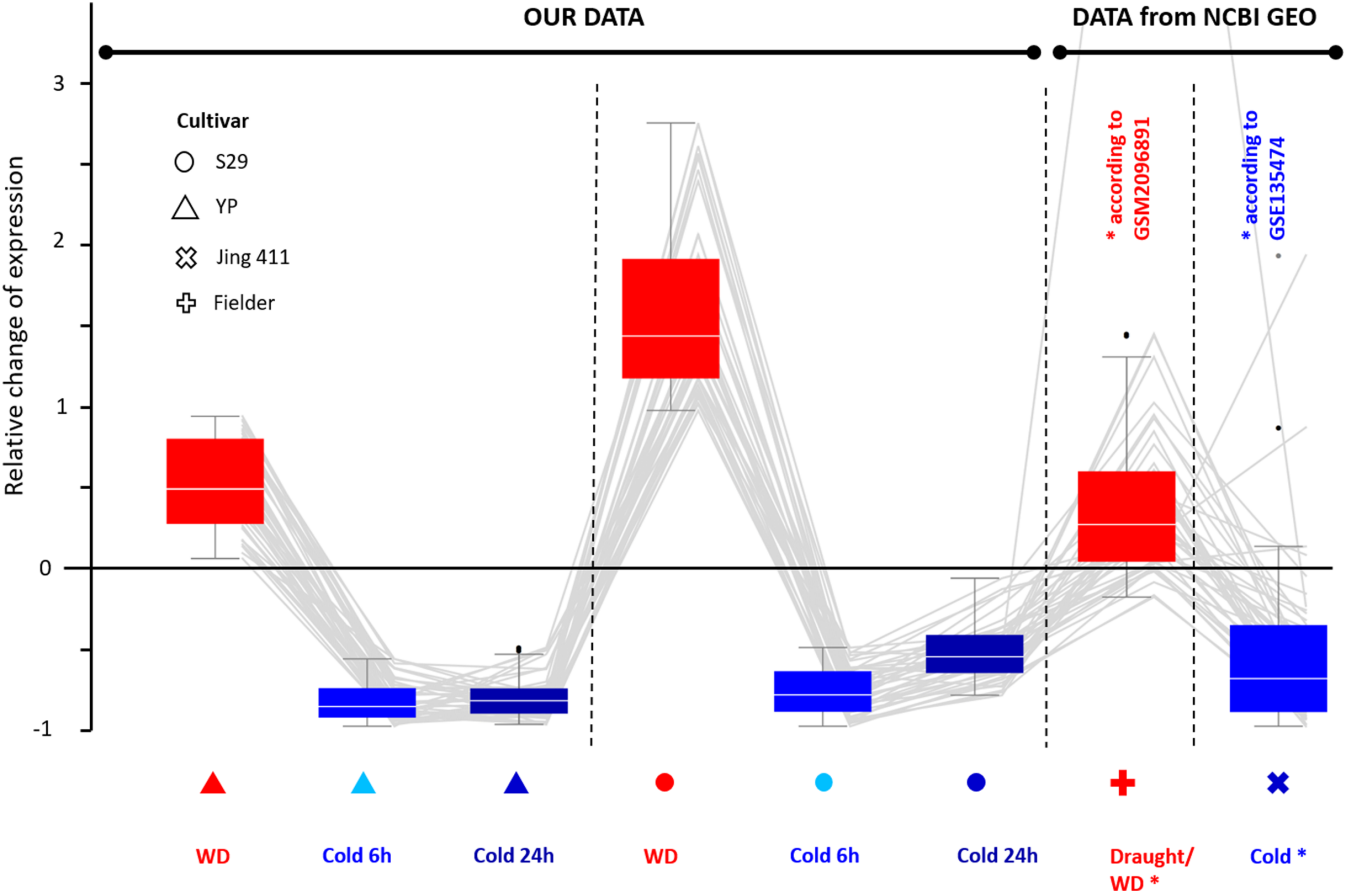
Differential expression for 43 wheat genes of cultivars YP, S29, Jing 411, and Fielder showing a similar pattern. Genes were identified by transcriptome analysis for varieties YP and S29 and confirmed the same expression pattern in varieties Jing 411, and Fielder in independent experiments. The boxes extend from the Q1 to Q3 quartile values of the data, with an orange line at the median (Q2). The whiskers extend from the edges of box to show the range of the data. The position of the whiskers is set by 1.5 × IQR (IQR = Q3 − Q1) from the edges of the box.

Comparing transcripts of evolutionarily distant varieties may help identify genes with a conservative expression pattern in response to stress for bread wheat. These genes may play a significant role in stress response. According to our data, we selected genes characterizing by equally directed expression changes in response to WD and cold. Then we compared the expression changes for the same genes of cv. Jing 411 under cold conditions (according to GSE135474) and data of cv. Fielder under drought (WD) conditions (according to GSM2096891) and found a high correspondence in the directions of expression changes. Thus, we identified 43 genes (the list is in File S10) up-regulated during WD and down-regulated during cold exposure; its relative changes in expression are shown in Fig. 4. There were eight Chaperone protein dnaJ genes, two transcription factor genes (WRKY and bZip), two genes involved in cell wall biosynthesis (Glucan endo-1,3-beta-glucosidase and Xyloglucan endotransglucosylase/hydrolase), two transmembrane proteins, two cell receptor, and DNA mismatch repair protein mutL gene. The functions of 26 genes are poorly studied.

### Comparison of DEGs with QTL data

To identify genes playing a crucial role in the stress response for the studied varieties, we compared the list of WD-induced DEGs revealed from our data with QTLs responsible for the antioxidant system activity under WD-stress. In leaves of inter-varietal single chromosome substitution line S29 (YP 2A), increased activity of superoxide dismutase (SOD) under the influence of water deficiency and lipoxygenase (LOX) at regular watering were showed (Osipova et al., 2020). Further personal discussions with the authors revealed that regions of the chromosome 2A at coordinates 102, 108, and 149 cM played a significant role in these processes. BLASTN indicated the coordinates for the markers on chromosome 2A of cv. CS flanking these regions (File S9). We identified 3,502 genes located between the coordinates of the markers in the regions of 102 cM (1819 genes), 108 cM (1567 genes), and 149 cM (116 genes). Eleven genes from this set revealed differential expression in response to WD (Table 2). The TraesCS2A01G438200 gene lowering its expression in response to drought for cv. S29 is a homolog of the gene encoding alternative oxidase 1A, which is involved in electron transfer in mitochondria and is involved in cold acclimatization of plants.

**Table 2:** The most significantly differentially expressed genes changing under WD-stress for cv. S29 and cv. YP and located in QTLs responsible for the antioxidant system activity under WD-stress indicated in Osipova et al. (2013) and Osipova et al. (2020). Arrows ↑ and ↓ mean the direction of significant change with E-value < 0.05 after Benjamini–Yekutieli correction.

QTL coordinates, cM	S29	YP	*T. aestivum* gene	*A. thaliana* ortholog	Description according to TAIR database
101–104	–	↑	TraesCS2A01G157400	AT2G02990	Encodes a member of the ribonuclease T2 family that responds to inorganic phosphate starvation, and inhibits production of anthocyanin. Also involved in wound-induced signaling independent of jasmonic acid. Its expression is responsive to both phosphate (Pi) and phosphite (Phi) in roots.
	–	↑	TraesCS2A01G158800	AT2G38900	Predicted to encode a PR (pathogenesis-related) peptide that belongs to the PR-6 proteinase inhibitor family. Six putative PR-6-type protein encoding genes are found in Arabidopsis: At2g38900, At2g38870, At5g43570, At5g43580, At3g50020 and At3g46860.
	–	↑	TraesCS2A01G222500	AT1G61930	Senescence regulator (Protein of unknown function, DUF584)
	↑	↑	TraesCS2A01G167300	AT1G72310	Encodes a putative RING-H2 zinc finger protein ATL3 (ATL3).
	↓	–	TraesCS2A01G161700	AT3G22800	Leucine-rich repeat (LRR) family protein
	↓	–	TraesCS2A01G209700	–	–
	↓	↓	TraesCS2A01G235000	–	–
108	–	↑	TraesCS2A01G389400	AT2G46680	Encodes a putative transcription factor that contains a homeodomain closely linked to a leucine zipper motif. Transcript is detected in all tissues examined. It is transcriptionally regulated in an ABA-dependent manner and may act in a signal transduction pathway which mediates a drought response.
	↓	–	TraesCS2A01G438200	AT3G22370	Encodes AOX1a, an isoform of alternative oxidase that is expressed in rosettes, flowers, and root. The alternative oxidase of plant mitochondria transfers electrons from the ubiquinone pool to oxygen without energy conservations. It is regulated through transcriptional control and by pyruvate. Plays a role in shoot acclimation to low temperature. Also is capable of ameliorating reactive oxygen species production when the cytochrome pathway is inhibited. AOX1a also functions as a marker for mitochondrial retrograde response. The mRNA is cell-to-cell mobile.
149	↑	–	TraesCS2A01G544700	–	–
	↑	–	TraesCS2A01G547300	–	–

Zhao et al. (2020) revealed significant SNPs associated with cold resistance which were scattered over 18 chromosomes, resulting in 361 candidate genes. Among them we identified 7 genes significantly changing expression in response to long and short cold exposure for the studied varieties (Table 3). Among them, three genes (TraesCS5A01G310100.1, TraesCS7D01G401500.1, and TraesCS7B01G320100.3) respond only to 24h-cold exposure for cv. YP, while two genes (TraesCS3D01G019300.1 and TraesCS6D01G016900.1) showed an increase in expression only for one of the studied varieties.

**Table 3:** The most significantly differentially expressed genes changing under cold stress for cv. S29 and cv. YP and also identified in GWAS and experimental analysis of Zhao et al. (2020). Arrow ↑ means the direction of significant change with E-value < 0.05 after Benjamini–Yekutieli correction.

S29	YP	S29	YP	*T. aestivum* gene	*A. thaliana* ortholog	Description according to TAIR database
**cold 6 h**		**cold 24 h**				
–	–	–	↑	TraesCS5A01G310100.1	AT4G25480	Encodes a member of the DREB subfamily A-1 of ERF/AP2 transcription factor family (CBF3). The protein contains one AP2 domain. There are six members in this subfamily, including CBF1, CBF2, and CBF3. This gene is involved in response to low temperature and abscisic acid.
–	–	–	↑	TraesCS7D01G401500.1	AT4G19390	Uncharacterized protein family.
–	–	–	↑	TraesCS7B01G320100.3	–	–
–	↑	–	↑	TraesCS3D01G019300.1	AT3G62160	HXXXD-type acyl-transferase family protein
↑	–	↑	–	TraesCS6D01G016900.1	AT1G21230	Encodes a wall-associated kinase The mRNA is cell-to-cell mobile.
↑	–	–	↑	TraesCS5D01G355500.1	AT1G33110	MATE efflux family protein
–	↑	↑	↑	TraesCS2D01G425200.1	–	–

### Assessment of stress-induced changes in expression coherence between homeoallelic gene sets

**Figure 5:**
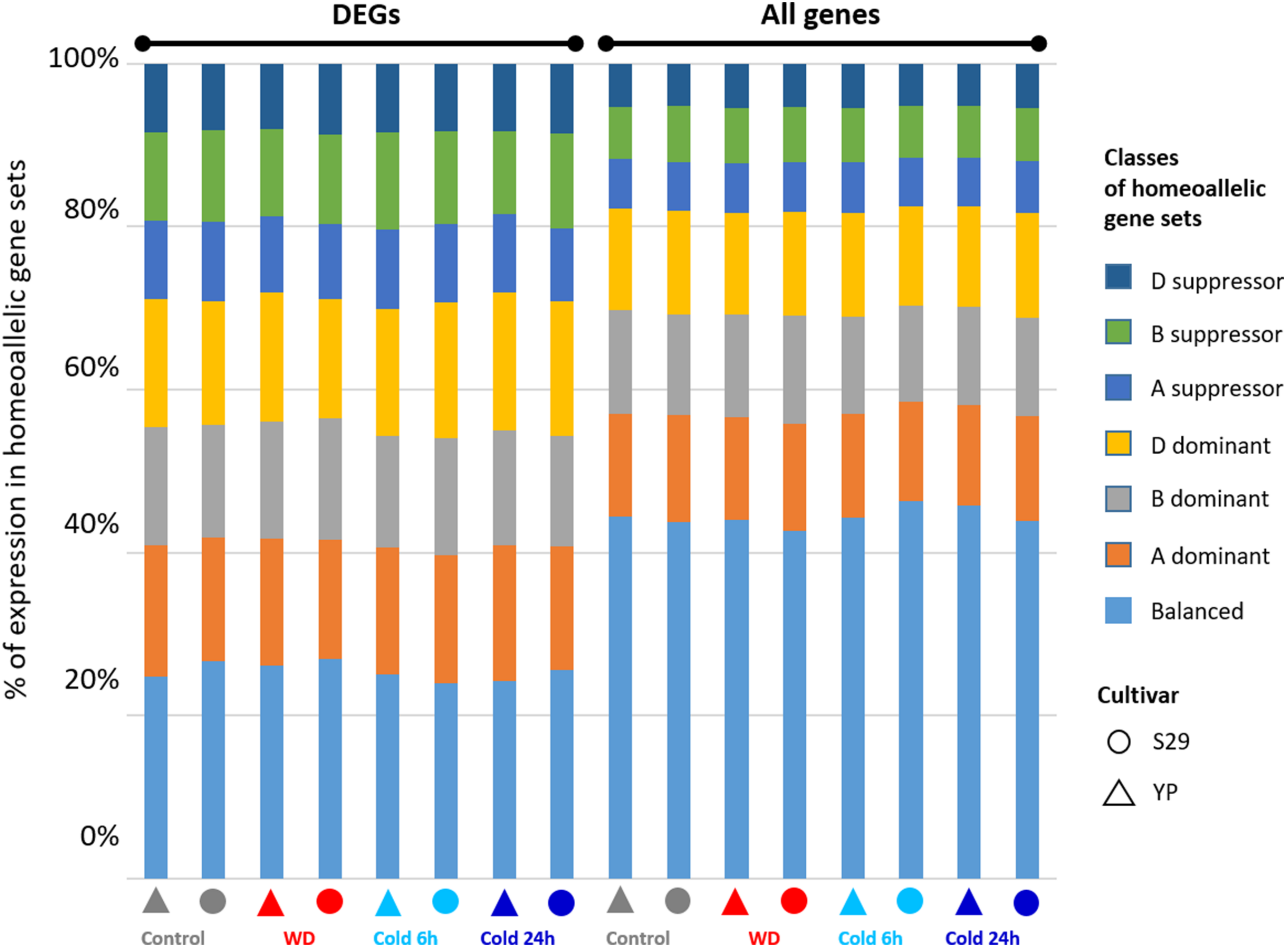
Distribution of the ratios for homeoallelic gene sets divided into seven classes (A- B- or D-dominant, A- B- or D-suppressor and balanced) described in (Gálvez et al., 2019) and determined from the data on gene expression under control, cold (6 h and 24 h), and WD conditions for the complete set of genes and the DEGs-subset.

The species’ genomic constitution is an essential factor to consider when studying gene expression changes in response to environmental stimuli. Hexaploid wheat has homeoallelic sets of genes for which regulation may be more or less coordinated. Previously this effect was observed for the cv. TAM107 while studying the response to high temperature and drought (Liu et al., 2015). For cv. S29 and cv. YP, we performed a similar comparative analysis (Fig. 5) of the expression consistency within homeoallelic gene sets. Then we compared the results for all homeoallelic gene sets and those sets, including at least one DEG in response to WD and cold stress.

To identify homeoallelic protein-coding genes of wheat, we compared all protein sequences using BLASTP based on the URGI database (Alaux et al., 2018). Triplets of genes assigned to homoeologous chromosomes from different subgenomes were assembled into homeoallelic sets. Protein-coding genes with an unspecified location in the genome were removed from further analysis. A total of 21,443 homeoallelic gene sets were identified; among them, 1599 sets showed differential expression for at least one of the homoeologs, according to our data (see Files S5 and S6 for details).

The homeoallelic gene sets were divided into seven classes: A- B- or D-dominant, A- B- or D-suppressor, and balanced according to the algorithm described in Materials and methods. For the complete set of genes and the DEGs-subset, we revealed the difference in the ratios of these classes (Fig. 5) for the data on gene expression under control, WD, and cold (6 h and 24 h) conditions (corresponding complete numerical data are given in File S6). For all homeoallelic gene triplets, about 44.4% of sets have balanced expression. For each of the subgenomes, one gene from the triplet dominate in about 12.5% of sets, and is suppressed in about 6% of sets. In contrast, among triplets showing differential expression in response to stress at least for one gene, the proportion of balanced triplets is lower (25%), and the fractions for other classes increase uniformly. Thus, we can conclude that for cv. S29 and YP, gene expression changes under WD and cold stress cause an increase in the heterogeneity of expression values within homeoallelic gene sets.

### SNP distribution analysis

To involve the new genetic material of the studied varieties in genomic selection, it is also necessary to expand genotyping possibilities using transcriptome sequencing data, which allows identifying SNPs in transcripts. The differences in the sequences between the reads for cv. YP and cv. S29 and the reference genome of cv. Chinese Spring (CS) revealed the distinguishing SNPs. A total number of 27,168 SNPs were found, of which 16,472 for cv. YP, 17,977 for cv. S29, and 7,281 (approx. 26.8%) for both cultivars. A detailed description of the mutation number is given in Files S7 and S8.

**Figure 6:**
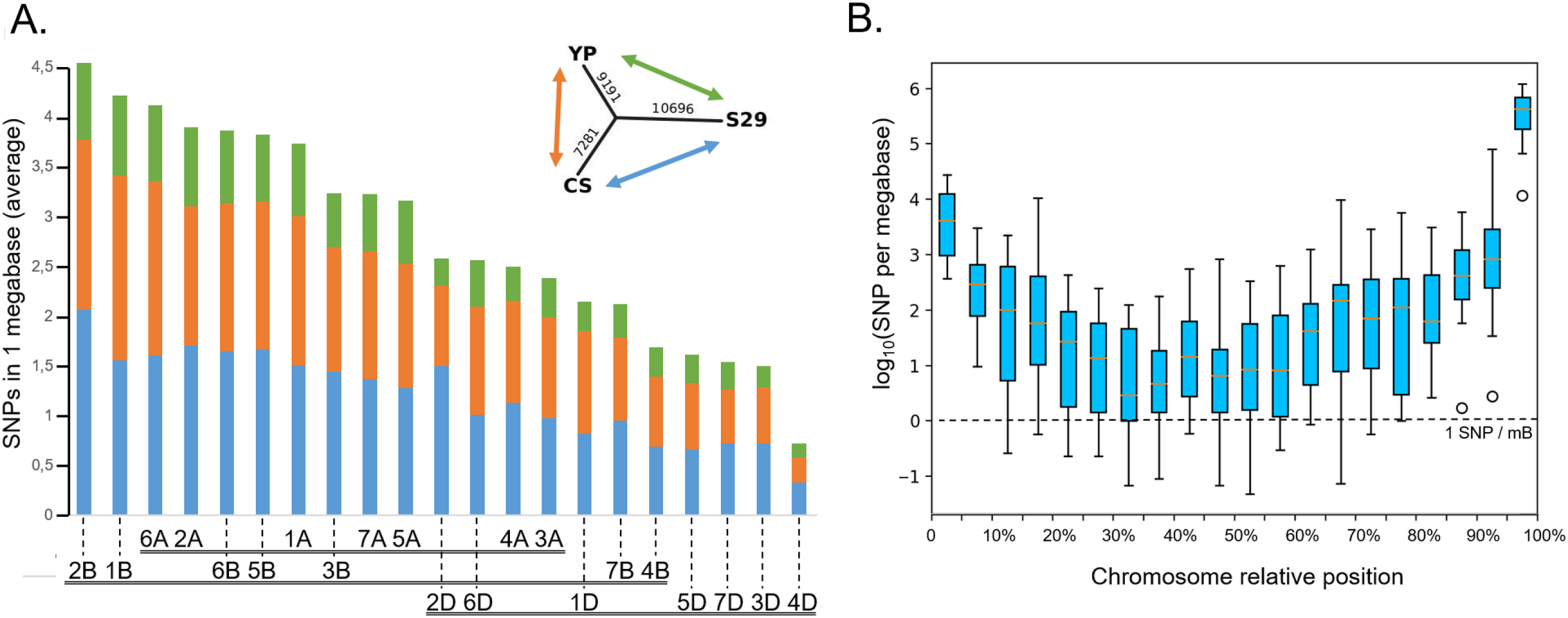
Distribution of SNPs between chromosomes. (A) The average amount of SNPs per 1 MB per chromosome, distinguishing the varieties. The incut shows the dendrogram reflecting the total number of SNPs differentiating varieties. The colors show between which varieties the differences were considered (green is for S29↔YP, orange is for CS↔YP, blue is for S29↔CS). (B) The distribution of SNPs along the averaged chromosome. The boxes extend from the Q1 to Q3 quartile values of the data, with an orange line at the median (Q2). The whiskers extend from the edges of the box to show the range of the data. The position of the whiskers is set by 1.5 × IQR (IQR = Q3 − Q1) from the edges of the box.

On average, each chromosome of the studied species carries 800 SNPs, chromosome 2A has the most of them (1,092 for cv. YP and 1,333 for cv. S29), as well as chromosome 2B (1,369 for cv. YP and 1,653 for cv. S29), while chromosome 4D carries the least SNP number (129 for cv. YP and 167 for cv. S29). For most chromosomes, the number of detected SNPs increases in telomeric regions (Fig. 6B and File S8) 84% of all mutations are of types “3′ prime UTR variant” (32%), “downstream gene variant” (34%), and “synonymous variant” (18%). Another 14% falls on “intron variant” (6%), “missense variant” (4%), and “synonymous variant” (4%).

Non-synonymous substitutions in the coding parts of genes were considered in more detail. They represent 0.12% of the total number of SNPs. Such polymorphisms were identified in 95 genes for cv. YP and in 90 genes for cv. S29, and in 27 genes for both varieties. The described genetic polymorphisms can be used in the future to develop molecular markers for genetics and breeding.

## Discussion

Drought and low temperatures are widespread types of abiotic stresses leading to a drop in yield and wheat grain quality. Understanding the genetic mechanisms of response to these factors is crucial in the breeding of resistant varieties. The difference in stress response at the transcriptome level between resistant and susceptible cultivar has been studied (Hu et al., 2018) previously. However, the more important thing is distinguishing between general and variety-specific genetic mechanisms of the stress response. This work studied the full-transcriptome changes in response to abiotic stresses for two contrast bread wheat cultivars. Thus, German cv. YP is sensitive to stresses, particularly drought, while Russian cv. S29 is a drought-tolerant variety developed for arid steppe regions (Kumakov, Evdokimova & Buyanova, 2001). Cv. S29 cultivated in various agroclimatic areas is widely used in breeding and is characterized by the high quality of flour (Laikova et al., 2004). The data obtained in this work represent the first transcriptome profiling for the studied bread wheat varieties, which are actively used in breeding works and genetic researches.

According to our data for leaf tissues, the studied cultivars revealed a relatively low intersection of the DEGs lists (less than 10% for each type of stress, see Fig. 3A–3C), while according to (Hu et al., 2018) for wheat roots, the lists of DEGs overlap by 35% between the drought-tolerant cv. JM-262 and the sensitive cv. LM-2. It may be due to significant differences in stress-induced regulation between these varieties or due to a substantial change in gene expression in the leaf in response to stress. However, our data revealed that cv. S29 has more DEGs associated with WD-response and fewer DEGs associated with cold-response than cv. YP.

Among genes encoding the antioxidant system’s enzyme, Glutathione S-transferase and Glutaredoxin showed the most significant change in expression for both studied cultivars and stress factors. Glutaredoxin shows a decrease in the expression of the most gene copies, consistent with other experiments (Díaz et al., 2019). It was shown for arabidopsis that glutaredoxin is an essential element in the abiotic stress response, and it has many copies with multidirectional changes in gene expression (Zuther et al., 2004).

Dehydration responsive element-binding (DREB) transcription factors play a crucial role in plant growth, development, and stress responses; therefore, we tested all 210 genes from the DREB family indicated by Niu et al. (2020). Among them, we identified 12 genes as DEGs for cv. S29 and cv. YP. While only TraesCS7B01G028700 increased its expression in response to WD for cv. S29 confirming data from (Amalraj et al., 2016). The remaining 11 genes changed their expression in response to 24 h of cold treatment for cv. YP. The DREB3 gene studied by Shavrukov et al. (2016) was not identified as a DEG in our experiments.

We compared transcriptome profiles for the two studied cultivars and revealed groups of genes with a similar pattern in the expression change (Fig. 4). Among them, forty-three genes showed the highest degree of similarity in the transcriptional response to WD and cold stress. Though the studied varieties are quite evolutionarily distant genotypes, we also tested the expression pattern’s stability by comparing them in our experiment and the data obtained for other varieties under similar experimental conditions (Fig. 4) and revealed a similarity of the response. Found genes are presumably participants in the underlying regulatory pathways, which may be shared for a wide range of bread wheat genotypes. Among the identified genes, eight were annotated as Chaperone (dnaJ), which protects proteins from irreversible aggregation during synthesis (File S10). In functional profiling using g:Profiler (Raudvere et al., 2019), three significant terms were identified (GO:0005365-myo-inositol transmembrane transporter activity GO:0005366-myo-inositol: proton symporter activity GO:0015166-polyol transmembrane transporter activity) associated with transmembrane transport of polyols and inositol in particular (Fig. S11). For plant organisms, polyols also contribute significantly to symplastic and apoplastic transport of carbon and energy (Noiraud, Maurousset & Lemoine, 2001), especially in response to salt stress and drought. Inositol and related molecules are essential elements in maintaining osmotic homeostasis in plant and animal cells (Schneider, 2015), as well as precursors of various signaling molecules. In plants, inositol derivatives are also useful osmolytes that protect cell structures during drought, cold, and salt stress (Chauhan et al., 2000; Lott, Greenwood & Batten, 2017; Sengupta et al., 2008). Plants often accumulate myo-inositol in leaves in response to stressful effects (Nelson, Rammesmayer & Bohnert, 1998), which stimulate sodium uptake in leaves (Nelson, Koukoumanos & Bohnert, 1999) and in general, it can be a useful osmolyte in response to various stress factors. Assessment of the associative map of GO terms (Fig. S11) for all GO terms for 43 genes (conducted with amiGO 2 visualization tool (Carbon et al., 2009)) as the main groups of processes revealed active transcriptional regulation (File S10). In particular, it includes various inorganic ions, active metabolism, and transport of sugars associated with cell wall metabolism and active ion transport, which is also possibly associated with maintaining ionic homeostasis and inositol transport, since it has two main types: proton and sodium symporter.

For cv. S29 and YP grown under normal conditions, about 45% of the sets of homeoallelic genes exhibit consistent expression (Fig. 5 and Files S5 and S6). If we consider a subset of groups of homeoallelic genes where at least one gene differentially expressed in response to WD or cold exposure, this value will decrease to 25%. Thus, genes associated with stress response belong homeoallelic gene sets characterized by less similar expression values between them. These data are consistent with homeoallelic genes’ behavior during drought and elevated temperatures, where the proportion of such genes was 32% (Liu et al., 2015). Thus, it has been shown that a change in the regulation of gene expression in response to abiotic stress reduces uniformity in the homoeologous gene expression. This phenomenon is found in various wheat varieties and may indicate the subfunctionalization of genes inside homoeologous groups in response to various stresses (Osborn et al., 2003).

Cv. S29 and YP have only about 25% of the total mutations that distinguish both genotypes from cv. CS. (Fig. 6A, Files S6, S7). According to the estimated polymorphisms, all three varieties are located at an almost equal distance from a common ancestor (Fig. 6A, dendrogram) and do not have detectable intersecting chromosome regions. The number of SNPs grows from the centromeric region to telomeric regions faster than the number of genes (Choulet et al., 2014), indicating a relatively higher polymorphism of genes in these areas.

The distribution of mutations through the genome is uneven; the smallest number of polymorphisms is detected for D-genome’s chromosomes. On average, the proportion of mutations on D-chromosomes in homoeologic sets is 18%. Noticeably few mutations were detected for 3D, 4D, and 5D chromosomes. For wheat cultivars Exonerate, Nullitetra, and Kansas such a phenomenon was not observed (Winfield et al., 2016). About 97% of the identified SNPs are in a homozygous state. These results can be used to develop SNP and CAPS markers (Zhernakov et al., 2017).

## Conclusions

This study presents the first full transcriptome profiling of gene expression for stress-resistant (cv. S29) and stress-susceptible (cv. YP) wheat varieties under normal conditions, water deficiency, short (6 h) and long (24 h) cold stress (+4 °C) at the stage of the fourth leaf. For the first time for a polyploid organism, we used MACE technology for transcriptome analysis. It allowed us to identify 2,159 genes differentially expressed in response to stress and described subsets for varieties and stress factors. Based on a functional analysis of gene groups, we assume that several mechanisms are involved in the stress response, including changes in the photosynthetic apparatus, transcription factors (mostly WRKY), and zinc transporters. Under WD conditions, both varieties demonstrate a decrease in gene expression related to the photosynthetic apparatus (RuBisCO small, RbcS, Chloroa b-bind). Moreover, in cold conditions, the expression of genes associated with chloroplast and thylakoid membranes decreases. We identified 21 genes in QTLs associated with the antioxidant system’s activity under WD conditions. These genes significantly change expression values under WD conditions for the studied varieties. It was revealed that, in response to cold, a higher number of genes change expression than in the case of WD. Most homoeologous genes do not show a consistent response to stress. Also, we identified SNPs between cv. CS and the studied varieties and described their distribution across the genome. They will allow developing new molecular markers and involving these cultivars in high-level genetic work.

Thus, the integration of stress-induced transcriptome changes data for various varieties provided valuable information on the regulatory networks that underlie the plant response to stresses caused by cold and water deficiency. Further studies of wheat varieties that originated from different geographical zones will allow a deeper understanding of its adaptation to low temperatures and water deficiency.

## Supplemental Information

10.7717/peerj.11428/supp-1 Supplemental Information 1 Comparative characteristics of the moisture content of the substrate in the experiment creating water deficiency conditions.

10.7717/peerj.11428/supp-2 Supplemental Information 2 The number of reads sequenced and mapped with STAR to iwgsc v1 genome for all libraries.

10.7717/peerj.11428/supp-3 Supplemental Information 3 Fold changes for DEGs and the significance of the change and the reliability (after Benjamini-Yekutieli) of the change in expression in stress.

10.7717/peerj.11428/supp-4 Supplemental Information 4 Expression values for all obtained *Triticum aestivum* L. genes.

10.7717/peerj.11428/supp-5 Supplemental Information 5 The separation of homeoallelic gene sets according to expression consistency.

10.7717/peerj.11428/supp-6 Supplemental Information 6 Average expression values for homeoallelic gene sets.

10.7717/peerj.11428/supp-7 Supplemental Information 7 Identified SNPs for *Triticum aestivum* L. cv. YP and cv. S29 according to the type of mutation.

10.7717/peerj.11428/supp-8 Supplemental Information 8 Identified SNPs for *Triticum aestivum* L. cv. YP and cv. S29 by localization on the chromosome.

10.7717/peerj.11428/supp-9 Supplemental Information 9 QTL-marker positions on chromosome 2A.

10.7717/peerj.11428/supp-10 Supplemental Information 10 A list of 43 genes with a high degree of similarity of the transcriptional response to stress in evolutionarily distant varieties.

10.7717/peerj.11428/supp-11 Supplemental Information 11 GO enrichment in terms in the list of 43 genes with a high degree of similarity of the transcriptional response to stress in evolutionarily distant varieties.

## Abbreviations

The following abbreviations are used in this manuscript:

S29: Saratovskaya 29
YP: Yanetzkis Probat
CS: Chinese Spring
MACE: Massive analysis of cDNA ends
DEG: Differentially expressed genes
CAPS: Cleaved Amplified Polymorphic Sequences
SNP: Single Nucleotide Polymorphism
QTL: Quantitative trait loci
PCA: Principal component analysis
WD: Water deficiency
WDR: Water deficiency response
FPKM: Fragments Per Kilobase Million
FDR: False discovery rate
HMM: Hidden Markov Model
SOD: Superoxide dismutase
LOX: Lipoxygenase
POD: Peroxidase
CAT: Catalase
RuBisCO: Ribulose-1,5-bisphosphate carboxylase/oxygenase
DREB: Dehydration responsive element-binding.

## Additional Information and Declarations

### Data Availability

The following information was supplied regarding data availability: The data is available at NCBI: PRJNA630059.
