# Supplementary figures and images for: Comparative transcriptome profiling of a resistant vs susceptible bread wheat (Triticum aestivum L.) cultivar in response to water deficit and cold stress
Source: PeerJ. 2021 May 12;9:e11428. doi: 10.7717/peerj.11428 (PMC8123233; doi:10.7717/peerj.11428)

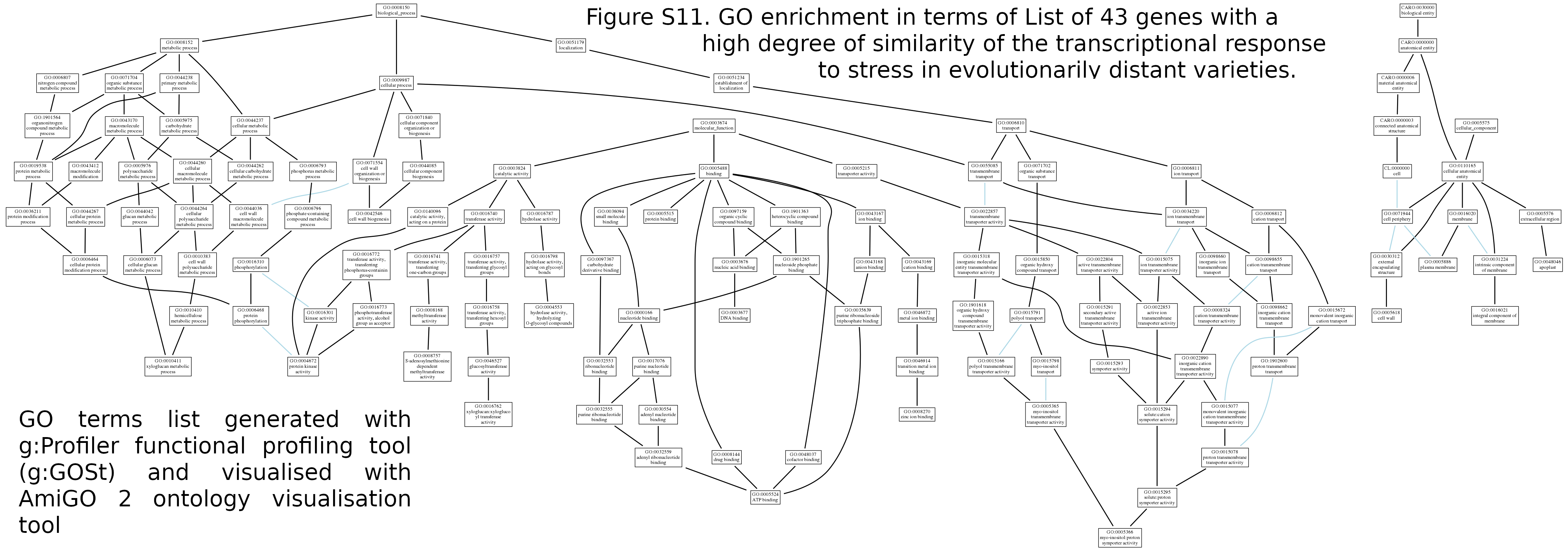

Supplement: Supplemental Information 11 [file peerj-09-11428-s011.png]
